# The Evolutionary Pathway of X Chromosome Inactivation in Mammals

**Published:** 2013

**Authors:** A.I. Shevchenko, I.S. Zakharova, S.M. Zakian

**Affiliations:** Institute of Cytology and Genetics, Siberian Branch, Russian Academy of Sciences, Prospekt Lavrentyeva, 10, Novosibirsk, Russia, 630090; State Research Institute of Circulation Pathology, Rechkunovskaya Str., 15, Novosibirsk, Russia, 630055; Institute of Chemical Biology and Fundamental Medicine, Siberian Branch, Russian Academy of Sciences, Prospekt Lavrentyeva, 8, Novosibirsk, Russia, 630090

**Keywords:** mammals, X chromosome inactivation, Xist.

## Abstract

X chromosome inactivation is a complex process that occurs in marsupial and eutherian mammals. The process is thought to have arisen during the differentiation of mammalian sex chromosomes to achieve an equal dosage of X chromosome genes in males and females. The differences in the X chromosome inactivation processes in marsupial and eutherian mammals are considered, and the hypotheses on its origin and evolution are discussed in this review.

## INTRODUCTION

The class Mammalia (mammals) is divided into two subclasses: Prototheria (monotremes) and Theria. In turn, the infraclasses Metatheria (marsupial mammals) and Eutheria (placental mammals) are distinguished in the Theria subclass. The divergence between monotremes and marsupial mammals took place 166.2 million years ago; the divergence between marsupial and placental mammals occurred 147.7 million years ago [1].

The ontogenesis of female marsupial and placental mammals is accompanied by a unique epigenetic phenomenon, heterochromatization of one X chromosome (out of two) and inactivation of its transcription, which is maintained in cell generations [2, 3]. This mechanism is believed to have arisen due to the necessity of gene dosage compensation in heteromorphic sex chromosomes in individuals of the opposite sex. In the subclass Theria, sex is determined by two heteromorphic sex chromosomes, X and Y. Males have the XY combination of sex chromosomes, while females have the XX combination. Since the Y chromosome contains only several tens of genes, as opposed to the X chromosome that contains approximately a thousand genes, most genes in the X chromosome are represented as a single copy in males (XY) and two copies in females (XX). As a result of inactivation of a single X chromosome in females, only one gene copy of the X chromosome is transcriptionally active in individuals of both sexes; thus, approximately equal amounts of the products of X-linked genes are synthesized in cells. X chromosome inactivation occurs due to the effect of specific nuclear RN As and chromatin modifications that repress transcription and differ in marsupial and eutherian mammals [3, 4]. The evolution of X chromosome inactivation is discussed in this review.

## PHENOMENOLOGY OF X-INACTIVATION IN MAMMALS

**Monotremes use a mechanism different from X chromosome inactivation for dosage compensation**

**Fig. 1:**
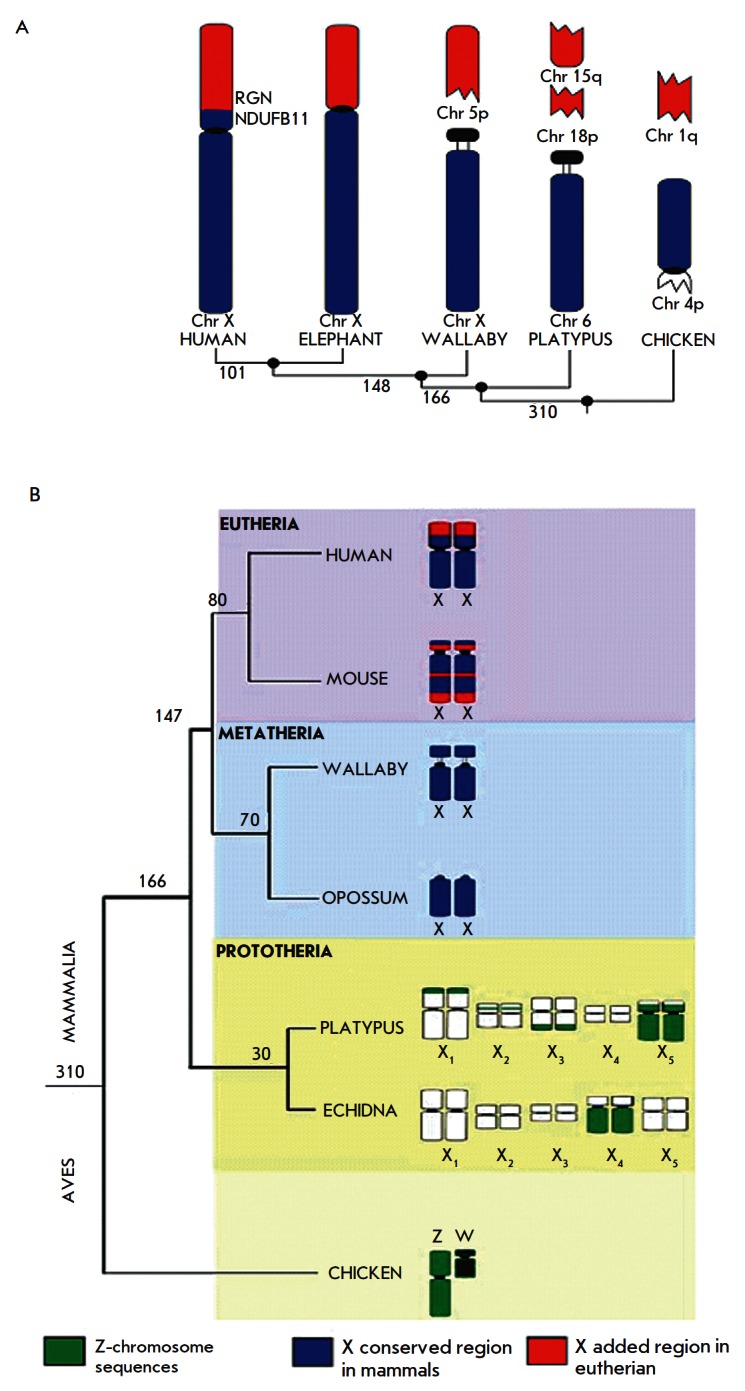
The origin and evolution of the mammalian X chromosome. А) Genes of the mammalian X chromosome have autosomal localization in birds (chicken) and monotremes (platypus, echidna). The X chromosome of marsupials (wallaby, opossum) represents the most ancient part of the mammalian X (shown in blue) and comprises 2/3 of the genes of the eutherian X chromosome. The eutherian X chromosome contains an added region (shown in red), which has autosomal localization in marsupials [7]. B) Monotremes have five X chromosomes, which show nothing in common with eutherian X but contain sequences homologous to the Z chromosome of birds [9]. The divergence time of the taxa (Mya) is shown on the branches of the phylogenetic tree

The living representatives of the most ancient mammalian subclass Prototheria, one platypus and four echidna species, are merged into the order of monotremes (Monotremata). Unlike the rest of mammals, the monotremes have a complex sex-determination system. The male platypus (* Ornithorhychus anatinus *) has five X and five Y chromosomes; five X and four Y chromosomes have been detected in male echidna (* Tachuglossus aculeatus *) [5–7]. The genes typical of the X chromosomes of marsupial and eutherian mammals have autosomal localization [7–9]. However, the genes typical of the sex chromosome Z of birds (including the *Dmrt1 *gene, which presumably plays the key role in sex determination in birds) have been found on the X chromosomes of monotremes. The most extensive region homologous to the chicken Z chromosome has been detected on the platypus X5 chromosome; less extensive regions of homology are localized on the X_1_, X_2_, and X_3_ chromosomes (*Fig. 1*).

**Table 1:** The ratio between the gene expression levels in the X chromosomes in female and male platypus cells and frequency of their monoallelic expression [10]

Chromosome	Gene	Ratio between the gene expression levels in females and males	Fraction of nuclei with monoallelic expression
Complete compensation			
X^1^	*Ox_plat_124086*	1.10	46
X^5^	ZNF474	1.01	53
X^5^	LOX	1.06	53
X^3^	APC	1.17	48
X^5^	SHB	1.23	53
Partial compensation			
X^5^	FBXO10	1.37	50
X^5^	EN14997	1.40	61
			
No compensation			
X^5^	SEMA6A	1.82	74
X^5^	DMRT2	2.04	47
X^5^	SLC1A1	2.78	45

All the X and Y chromosomes of monotremes contain homologous pseudoautosomal regions that enable conjugation between the X and Y chromosomes in meiosis [5–7]. However, the extensive regions of the platypus X_1_–X_5_ chromosomes (corresponding to ~12% of the genome) are nonhomologous and show no similarity to Y_1_–Y_5_. It is reasonable to expect that a mechanism of dosage compensation for the genes localized in these regions exists. A quantitative analysis of the transcription of the genes localized in the differentiated regions of different platypus X chromosomes [10] has demonstrated that some of them have identical transcription levels both in female and male cells, while expression of the remaining genes is either compensated partially or is not compensated at all (i.e., expression in female cells turns out to be twice as high as that in male cells) (*Table 1*). Thus, dosage compensation in monotremes presumably functions only for individual genes of the sex chromosome, resembling incomplete and variable dosage compensation in birds [11, 12]. In cell nuclei of female platypus, transcription of the genes exhibiting dosage compensation is revealed only for one of the homologous X chromosomes with a frequency of 50–70%. Nevertheless, total mRN A contains equal amounts of transcripts corresponding to each homologue. These data provide grounds for assuming that dosage compensation in monotremes occurs due to a decrease in the transcription level of one of the alleles (selected in each cell in a random manner) [10]. Since each pair of X chromosomes in female platypus has no visible distinctions in chromatin modifications at the cytological level, it is assumed that the dosage compensation in monotremes affects individual genes rather than chromosomes [13].

The pseudoautosomal region of the echidna X1 chromosome in some cell types is characterized by late replication [14], which can be regarded as an indicator of inactive chromatin, although the genes localized in this region are present both on X_1_ and Y_1_ and require no dosage compensation. Taking into account its susceptibility to inactivation, this region was previously regarded as an ancestral region when the mechanism of silencing of an entire chromosome could have presumably been formed. However, since the genes contained in this region in marsupial and eutherian mammals have autosomal localization and are not involved in inactivation, this assumption has been refuted.

Thus, it is an obvious fact that monotremes, unlike marsupial and eutherian mammals, use a mechanism that differs from X chromosome inactivation for dosage compensation.

**Table 2:** Status of gene expression in the X chromosomes in different marsupial species

Gene	Species	Method	Inactivation in somatic tissues
G6pd	Macropus robustus	Isoenzyme analysis, SNuPE	Complete
	Macropus rufogriseus	Isoenzyme analysis	Complete
	Didelphis virginiana	Isoenzyme analysis	Partial
	Monodelphis domestica	RT-PCR	Complete
Gla	Antechinus stuarttii	Isoenzyme analysis	Complete
	Kangaroo hybrids	«	Complete
Pgk1	Macropus giganteus	«	Tissue-specific
	Macropus parryi	«	«
	Trichosurus vulpecula	«	«
	Didelphis virginiana	«	«
	Monodelphis domestica	SNuPE	Partial

**Imprinted, incomplete and tissue-specific X chromosome inactivation in marsupial mammals**

Infraclass Metatheria (marsupials) comprises 270 species, 200 of which live in Australia; 69, in South America; and 1, in North America. The evolutionary segregation between Australian and American marsupials occurred 70 million years ago [9, 15]. The sex chromosomes in marsupial and eutherian mammals have a common origin. The X chromosome in marsupials represents 2/3 of the X chromosome of eutherian mammals; the remaining third of the genes are localized on the autosome (*Fig. 1*). Marsupials are the most ancient mammals; dosage compensation in female marsupials occurs due to X chromosome inactivation; however, the inactivation processes in marsupial and eutherian mammals differ significantly.

Nonrandom imprinted inactivation is typical of all marsupial tissues; it involves suppression of gene transcription and establishment of late replication in the S phase of the cell cycle, exclusively on the X chromosome inherited from the father [16, 17]. The untranslated nuclear RN A *Rsx *(RN A-on-the-silent X), which can propagate over the inactive X chromosome and repress gene transcription, is presumably responsible for the inactivation process at the chromosomal level [4]. The imprinted inactivation of three genes of the X chromosome has been studied in tissues of eight species (*Table 2*). It was found that the inactive status of the X chromosome inherited from the father is unstable, and that genes are frequently reactivated. It turns out that inactivation in marsupials does not affect all genes to the same extent (i.e., is incomplete). Moreover, the same loci of the X chromosome can be inactivated to different extents depending on a particular tissue. Thus, the phosphoglycerate kinase A (*Pgk1*) gene in the Virginia (North American) opossum *Didelphis virginiana* is completely inactivated in all tissues, whereas no stable repression of the paternal allele of the glucoso- 6-phosphate dehydrogenase (*G6pd*) gene is observed in most tissues [18]. In the gray short-tailed opossum*Monodelphis domestica*, unlike the Virginia opossum, the paternal *G6pd* allele is stably inactivated, whereas* Pgk1 *exhibits incomplete inactivation in all tissues [19]. Thus, orthological genes can be inactivated to different extents in different marsupial species.

It should be mentioned that X chromosome inactivation is not the only mechanism of dosage compensation in marsupials. In the members of the bandicoot family (Paramelidae), the Y chromosome in males and one of the two X chromosomes in females are eliminated at different ontogenic stages in somatic cells [20]. The elimination of sex chromosomes in different tissues can be observed either in all cells or in some of them. The investigation of the expression of the alleles of the X-linked *Pgk1 *gene in the southern brown bandicoot* Isoodon obesulus *shows that only the X chromosome inherited from the father is eliminated in females [21]. In the cells where sex chromosomes have not been eliminated, the X chromosome of paternal origin in females and the Y chromosome in males are late-replicating. The mechanism of elimination of sex chromosome is unknown; however, the preferential elimina tion of the X chromosome inherited from the father and asynchronous replication of the X chromosomes in females attest to the fact that this process emerged in marsupials as a trend in the evolution of the X chromosome inactivation process.

**Eutherian mammals have imprinted and random X chromosome inactivation, which are controlled by the inactivation center and the** *Xist* **gene**

Infraclass Eutheria (placental mammals), which is subdivided into the four superorders Afrotheria, Xenarthra, Euarchontoglires and Laurasiatheria, is the most numerous, diverse, and common mammalian infraclass. The X chromosome in eutherian mammals consists of the genes constituting the X chromosome in marsupials by 2/3 and contains an added region, which has autosomal localization in marsupials [9] (Fig. 1). As opposed to marsupial mammals, the X chromosomes of paternal and maternal origins are inactivated with equal probabilities in the cells of adult female eutherians; hence, on average half of the cells express the genes of the paternal X chromosome, while the remaining half express the genes of the maternal X chromosome. Unlike imprinted inactivation, random inactivation embraces most genes on the X chromosome and is stably maintained through cell generations. It should be mentioned that the genes in the added region of the X chromosome in eutherian mammals, which were localized on the autosome in marsupials and did not participate in the inactivation process, are inactivated with a lower efficiency and are capable of avoiding inactivation [22]. The random inactivation in eutherians comprises several stages: counting the number of X chromosomes per diploid genome, choice of an X chromosome for inactivation, initiation of activation, and propagation of the inactive status and its maintenance through cell generations [3, 23]. It is possible that the stage involving the choice of the X chromosomes (during which the mutually exclusive choices of the future active and inactive X chromosomes (like in a mouse) occurs) is typical not of all eutherian species. Thus, inactivation in early ontogenesis of the rabbit occurs stochastically, resulting in the formation of different cells, where 1) neither one of the X chromosomes is inactivated, 2) both X chromosomes are inactivated, or 3) one X chromosome out of two is randomly inactivated. Due to the disrupted gene dosage, the former two cell types subsequently die, while the remaining cells with normal inactivation form the organs and tissues of the organism [24].

In certain taxa of eutherian mammals (e.g., in rodents and artiodactyles), in addition to the random inactivation there also exists imprinted, incomplete and unstable inactivation of the X chromosome inherited from the father (however, this occurs exclusively at the pre-implantation stages of embryogenesis and remains in cells resulting in extraembryonic organs (placenta and vitelline sac) [25, 26].

Both the random and imprinted inactivation in eutherians are controlled by the inactivation center (XIC) and the *Xist *gene, which have not been detected in monotremes and marsupials [3, 23]. During the random inactivation, the *Xist *gene ensures initiation of inactivation and propagation of the inactive status, while the other elements of the inactivation center function at the stage of the counting of X chromosomes and choice of the chromosome to undergo inactivation.

**The evolution of complete and stable inactivation was accompanied by substitution of the noncoding RNA** *Rsx* **by** *Xist* **and the emergence of** *Xist* **-dependent modifications in the histones on the inactive X chromosome, along with DNA methylation in promoters**

**Fig. 2:**
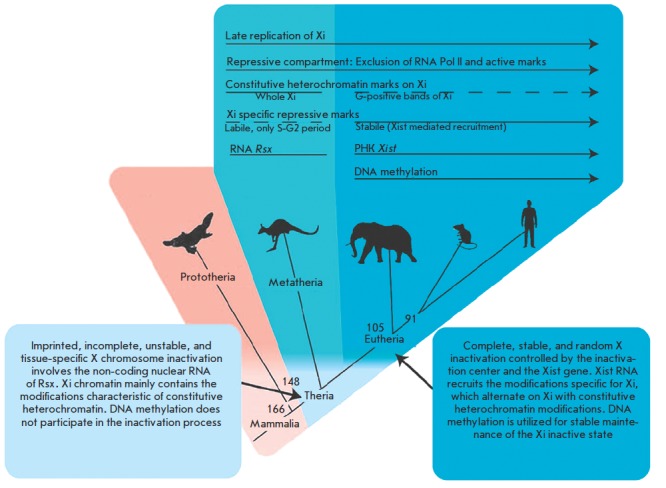
The evolution of the epigenetic mechanisms underlying X chromosome inactivation in mammals [28]. Xi is the inactive X chromosome. The divergence time of the taxa (Mya) is shown on the branches of the phylogenetic tree

Despite the differences, there are a number of common features between the X chromosome inactivation in marsupial and eutherian mammals, which presumably reflect the fundamental and the most ancient mechanisms underlying this process (*Fig. 2*) [13, 27, 28]. Both in marsupials and eutherian mammals, the inactive X chromosome is revealed in female interphase nuclei in the form of a cytologically discernible compact chromatin mass known as the Barr body. The DNA-dependent RN A polymerase II responsible for gene transcription is almost completely eliminated from the chromosomal area of the inactive X chromosome in interphase nuclei. The inactive X chromosome is late-replicating; during the replication stage, it migrates to the perinucleolar region of the nucleus, which is enriched in the enzymes required to reproduce the inactive chromatin structure. Covalent histone modifications typical of transcriptionally active chromatin are eliminated in the inactive X chromosome, while modifications typical of transcriptionally inactive chromatin are present. Chromatin of the inactive X chromosome contains untranslatable nuclear RN A, which is expressed only from the inactive X chromosome and propagates over it, resulting in gene inactivation.

**Fig. 3:**
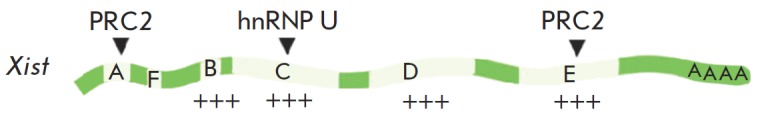
Functional RNA domains of the Xist gene. A, B, C, D, E, F – minisatellite repeats included in Xist RNA. (+++) – sequences responsible for Xist RNA spreading on the X chromosome. Arrows indicate the A- and E-repeat regions involved in binding of the PRC2 protein complex and the C-repeat region responsible for Xist RNA binding to the inactive X chromosome by the hnRNP U (SP120/ SAF-A) protein. A-repeats are also necessary for transcriptional gene silencing and organization of the inactive X chromosome compartment [3]

It should be emphasized that marsupial and eutherian mammals use completely different, unrelated in terms of their origin and nuclear noncoding RN As of *Rsx *and *Xist*, which exhibit similar properties and behavior during the inactivation process [4]. Both noncoding RN As are enriched in microsatellite repeats, which are significant functional domains required for the repression of transcription, propagation over the inactive X chromosome, and binding of the protein complexes responsible for chromatin modification (as has been demonstrated for *Xist *RN A) [29] (*Fig. 3*). The evolutionary conserved minisatellite A-repeats localized in the first exon of the *Xist *gene play a significant role in the inactivation of the transcription of X chromosome genes [30]. Deletion of the A-repeats renders* Xist *RN A incapable of inducing inactivation of the transcription of X-linked genes, although it can still normally propagate along the X chromosome [29, 31]. The propagation of *Xist *RN A along the X chromosome is controlled by the cumulative action of the microsatellite repeats B, C, D, and E [32]. The area of minisatellite C-repeats is responsible for the binding of Xist RN A to the chromatin of the inactive X chromosome via the hnRN P U protein, which is also known as SP120 and SAF-A (scaffold attachment factor A) [31, 33–35]. hnRN P U (heterogeneous nuclear ribonucleoprotein U) is a protein that contains three conserved domains: SAF-Box, which can bind to the AT-rich DNA region known as S/MAR (scaffold- or matrix-attachment region); the SPRY domain (Spla and Ryanodine receptor) with an unknown function; and the RN A-binding domain RGG (arginine-glycine-glycine). The presence of these domains makes it possible for hnRN P U to interact with *Xist *DNA and RN A, which facilitates its retention in the inactivated X chromosome [35].

It should also be noted that during the whole cell cycle the inactive X chromosome in marsupials is stably associated with heterochromatin protein HP1, histone H3 trimethylated at lysine K9, and histone H4 trimethylated at lysine K20, which are typical of the centromeric and telomeric regions of constitutive heterochromatin [13, 28, 36]. Some modifications specific to the inactive X chromosome in eutherians (e.g., histone H3 trimethylated at lysine 27) may temporarily emerge on the inactive X chromosome of marsupial mammals during the period between the S- to and the early G2- phase of the cell cycle.

In eutherians (similarly to marsupials), the repression of the entire X chromosome at the early stages of imprinted inactivation may occur exclusively as a result of the modifications typical of constitutive heterochromatic regions [37]. At the later stages of imprinted inactivation, as well as in the case of random inactivation, these modifications occur on the inactive X chromosome only in the regions enriched in repeats, which correspond to the G-positive bands. The regions of the inactive X chromosome enriched in genes are stably repressed during the whole cell cycle via the trimethylation of H3 at lysine K27, monoubiquitination of H2A at lysine K119, and insertion of the histone macroH2A1.2 (which are colocalized with *Xist *RN A) into chromatin [38–42]. The emergence of modifications capable of colocalizing with the *Xist *gene depends on its expression; repression of *Xist *and disturbances in the propagation of its RN A result in elimination of these modifications from the inactive X chromosome [29, 31, 43]. Moreover, it has been revealed that *Xist *RN A contains two sites that are capable of binding to the protein complex PRC 2 (Polycomb repressive complex 2), whose proteins function as histone methyltransferases responsible for the trimethylation of H3K27 [44].

Methylation of DNA in the inactive X chromosome is another epigenetic difference during inactivation in marsupial and eutherian mammals. The DNA of the inactive X chromosome in the embryonic tissues of eutherian mammals (as opposed to that of the active chromosome) is hypermethylated at the CpG dinucleotides localized in the promoters and 5’-untranslated regions of the genes during random inactivation [45]. The methylation is detectable during unstable imprinted inactivation neither in the extraembryonic tissues of eutherians nor in the somatic tissues of marsupials [18, 19, 46]. Methylation of promoter DNA during random inactivation has presumably emerged in eutherians as an additional stage of stabilization of the inactive status of the X chromosome in somatic cells.

## HYPOTHESES CONCERNING THE ORIGIN AND EVOLUTION OF X CHROMOSOME INACTIVATION

**Imprinted inactivation is likely to be more ancient**

Imprinted X chromosome inactivation, which occurs in all marsupial tissues and organs and in extraembyonic organs (placenta, vitelline sac) in a number of eutherian mammals, is considered to be the most ancient and primitive X chromosome inactivation. Imprinted inactivation has further evolved into the more preferable process of random inactivation as it incorporates the mechanisms of counting the number of X chromosomes per diploid set and choosing the future inactive X chromosome, which are controlled by the inactivation center.

Imprinted inactivation in certain eutherian taxa could have been retained or emerged again as it incorporated the new mechanisms offered by the inactivation center and the *Xist *gene. Thus, at least in mice, imprinted inactivation involves XIC and *Xist*. Imprinting preventing *Xist *expression and protecting the X chromosome inherited from the mother against inactivation has been detected in XIC [23]. However, imprinted inactivation has been completely eliminated in the other taxa (e.g., in humans) [47].

**The inactivation process could have originated from the mechanisms of imprinted or random monoallelic expression of autosomal genes and from meiotic silencing of sex chromosomes**

There is at present no satisfactory explanation for the origin of the X chromosome inactivation. The inactivation mechanism could have emerged *de novo *on the X chromosome or could have been borrowed from the existing silencing process.

There is a hypothesis that the mechanism that is used for imprinted monoallelic expression of the genes on one of the two homologous autosomes could underlie imprinted X chromosome inactivation [48]. Imprinting of gene expression on autosomes is a common conserved process among marsupial and eutherian mammals. Nuclear RN As, whose expression causes transcriptional gene silencing *in cis*, elimination of the modifications typical of active chromatin, and recruitment of the modifications specific to inactive chromatin, are involved both in autosomal genomic imprinting and in X chromosome inactivation in eutherian mammals. In eutherians, both these processes occur at the early stages of embryonic development, are retained in placenta, and lost in the embryo.

It should be mentioned that the randomly established monoallelic expression of autosomal genes is also a rather common phenomenon. Thus, the genes of immunoglobulins, factory receptors, T-cell receptors, and natural killer cell receptors exemplify the genes with monoallelic expression, which is determined stochastically. A number of genes with random monoallelic expression are characterized by asynchronous replication: they are early-replicating on one homologue and late-replicating on the other one during the S-phase of the cell cycle. The asynchronous replication of these genes is likely to take root during early development. Clusters of different genes with monoallelic expression localized on the same chromosome at a considerable distance from one another are characterized by equal replication times within the same homologue [49]. This fact provides grounds to assume that each homologue within a pair has its own specifically organized chromosomal area, which is similar to the region of the inactive X chromosome that can be cytologically detected in the interphase nuclei of marsupial and eutherian mammals as a compact chromatin mass known as the Barr body [23]. Thus, it is possible that X chromosome inactivation originates from the mechanism of stochastic monoallelic gene expression, with imprinting introduced later [50].

It has also been assumed that imprinted inactivation of the X chromosome inherited from the father either originates from meiotic inactivation of sex chromosomes in spermatogenesis or is its extension [18]. During spermatogenesis, meiotic inactivation of sex chromosomes at the pachytene stage of meiosis results in transcriptional silencing of sex chromosomes, giving rise to the sex body (XY body). The assumption of the fact that imprinted inactivation of the X chromosome may be related to the process of meiotic inactivation of sex chromosomes in spermatogenesis is supported by the data indicating that chromatin modifications identical to those formed during meiotic inactivation are formed during imprinted inactivation in marsupial mammals and at the early stages of imprinted inactivation in eutherians [37]. The tentative cognation between meiotic and imprinted inactivation provides grounds to believe that X chromosome inactivation could have occurred at the early evolutionary stages without the participation of nuclear noncoding RN A (and if this RN A did exist, it did not play the key role in transcriptional repression). This assumption is based on the data indicating that similar modifications ensuring chromatin repression are not specific to the inactive X chromosome in case of meiotic and imprinting inactivation but are typical of all the regions of constitutive heterochromatin in the genome, and that their emergence (at least on the eutherian X chromosome) is independent of *Xist *expression. Moreover, meiotic inactivation and the early stages of imprinted inactivation in eutherian mammals can successfully occur in the absence of *Xist *RN A, as well [51, 52]. In marsupials, meiotic gene repression in spermatogenesis is also independent of the *Rsx *gene, which is not expressed at this stage [4]. Thus, it can be assumed that the role of nuclear RN A in X chromosome inactivation could have originally consisted in organization of the specific chromosomal area or in relocation of the inactive chromosome to the perinucleolar compartment in order to ensure its replication (these processes occur with the immediate participation of *Xist *RN A) [53–55]. It was not until some time later that nuclear RN As started to be used directly for transcriptional repression and recruitment of the protein complexes repressing chromatin. However, one should bear in mind that the core histones (along with the epigenetic data regarding the transcriptional status of chromatin) are in most cases replaced by protamines as chromosomes are packaged in sperm cells, while methylation of the CpG islands employed for the inheritance of the inactive status in X-linked genes has not been detected [19]. Hence, it remains unclear how the inactive status of chromatin can be transmitted to the zygote. Furthermore, since the molecular mechanisms of both meiotic and imprinted inactivation remain unknown, it is difficult to determine the actual cognation between these processes.

## ORIGIN AND EVOLUTION of the X INACTIVATION CENTER AND THE Xist GENE

**The genes of the X inactivation center originate from the protein-coding genes and mobile elements**

**Fig. 4:**
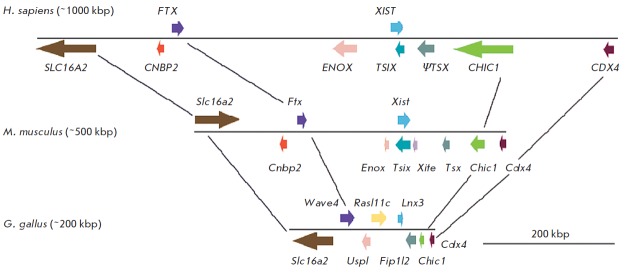
Comparison of the human and mouse X inactivation centres with its homologous region in chicken. Colored boxes represent genes; arrows show their transcription direction. Homologous genes in different species are shown in the same color. Lines connect the same homologous genes in the cognate loci of chicken, mouse and human. Cdx4, Chic1 and Slc16a2 are the conserved protein-coding genes that flank both eutherian XIC and its homologous locus in chicken. Cnbp2 is a protein-coding gene, which was retrotransposed to the XIC locus in the eutherian lineage. Tsx is a testis-specific protein-coding gene which partially evolved from the cognate chicken protein-coding gene Fip1l2. Note that human TS X is no longer functional and represents a pseudogene. Xist, Enox (Jpx) and Ftx are the genes of XIC-produced nuclear RNA; they show homology to the cognate chicken protein-coding genes Lnx3, Uspl and Wave4, respectively. The remainder of the chicken protein-coding gene Rasl11c is found in eutherian XIC between the genes Rtx and Enox (Jpx)

**Fig. 5:**
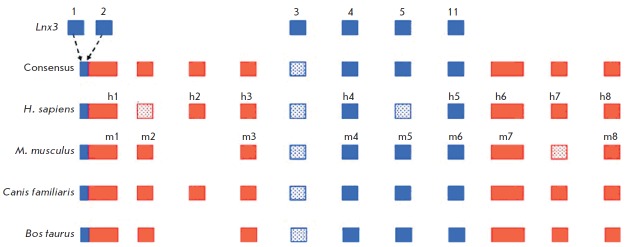
The origin of the Xist gene from the sequences of the protein-coding gene lnx3 and various classes of mobile elements [61]. Blue rectangles denote the exons that evolved from the gene Lnx3; red rectangles denote the exons originating from mobile elements; hatched blue and red rectangles denote the exon sequences detectable in the genome but not contained in the Xist transcript in the corresponding species. Consensus is a putative ancestral structure of the Xist gene. Exon numbering is given for the human (Homo sapiens) and mouse (Mus musculus) Xist genes: m1–m8 for mouse and h1–h8 for human

The X inactivation process in eutherian mammals is controlled by a complex genetic locus of the X chromosome, the X inactivation center (XIC). Along with* Xist*, the XIC of evolutionarily distant eutherian species contain two more genes that encode nuclear RN As –* Enox *(*Jpx*) and *Ftx*; it has been shown in experiments on mice that these genes activate *Xist *expression [56–59]. The XIC also contains the protein-coding genes* Tsx *and *Cnbp2*, whose products are not involved in inactivation [56]. It has been demonstrated that several protein-coding genes in the region of synteny with the XIC on chicken chromosome 4 exhibit homology with the genes of the inactivation center and could be their ancestors [60]. The Lnx3 gene, whose protein product contains the ubiquitin-ligase domain PDZ, underlies the formation of *Xist *(*Fig. 4*). It has been shown by comparing these genes that the promoter region and at least three exons of the *Xist* gene originate from the sequences of the *Lnx3 *gene. The largest first exon of the *Xist *gene presumably descended from endogenous retroviruses, whose fragments (after having been inserted into the locus) were amplified, producing simple tandem repeats of several types, which have been identified within it. The remaining exons of the *Xist* gene are syntenic to mobile elements of various classes (*Fig. 5*) [61]. The protein-coding genes surrounding the* Lnx3 *gene produced the other genetic elements of the inactivation center in mammals (* Fig. 4 *). The *Tsx *gene descended from the *Fip1l2 *gene. The two other genes,* Uspl *and *Wave4*, gave rise to *Enox *(*Jpx*) and *Ftx*, respectively. It can be noted that the *Enox *(*Jpx*) gene (as well as *Xist*) contains exons descending from mobile elements, which correspond to various types of repeats in different species [56, 57, 61].

**Monotremes and marsupials have no** *Xist* **gene; the region homologous to the X inactivation center in eutherians is separated by chromosomal rearrangements**

No direct orthologues of the Xist gene or other XIC sequences have been detected in monotremes and eutherians as a result of screening of the genome libraries and of a thorough search for homology in the sequenced genomes [62]. Moreover, protein-coding genes ancestral to XIC separated by independent chromosomal partitions and localizing as two individual groups (on the X chromosome in marsupials and on chromosome 6 in monotremes) have been detected in them [60, 62–64].* Lnx3 *RN A in marsupials has a native reading frame, is expressed both in males and females, and obviously functions as a protein-coding gene rather than as an untranslated nuclear RN A that is similar to *Xist*. Thus, protein-coding genes ancestral to XIC were transformed into the genes of the inactivation center only in eutherian mammals; the inactivation process in marsupials involves neither *Xist *nor XIC. The *Rsx *gene in marsupial mammals, which presumably has functions similar to those of the *Xist *gene in eutherians, flanks the protein-coding gene *Hprt *of the X chromosome and does not share a common origin with *Xist *and XIC [4]. ;

**The** *Xist* **gene and the X inactivation center rapidly accumulate speciesspecific differences during evolution**

**Fig. 6:**
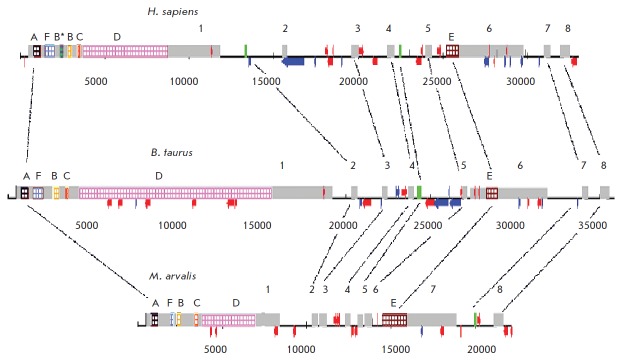
Comparison of the Xist gene structures in vole M. arvalis, B. taurus and H. sapiens. Grey rectangles represent exons (1–8). Green rectangles indicate parts of introns, which are exons in the Xist of other species. Lines connect the homologous sequences. Colored rectangles indicate arrays of tandem repeats, named A, B, C, D, E and F, which are present in the Xist exons of allthree species, and B*-repeats specific for humans. Yang species-specific LINE and SINE (short interspersed nuclear elements) mobile elements are indicated by blue and red arrows, respectively

The *Xist *gene has been detected in the genomes of representatives of all four mammalian superorders, including the most ancient Afrotheria and Xenarthra [62]. However, the *Xist *gene is not conserved and evolves very rapidly [56, 60, 61, 65]. The exons of the* Xist *gene evolve slower than introns do. The most conserved, exon 4, bears the best resemblance with the exon of the *Lnx3 *gene. Paradoxically, the first exon with some functions (and, in particular, the A-repeat region required to establish transcriptional gene silencing) evolves quicker than exon 4, whose deletion has no effect on inactivation. The number of exons per gene in different eutherian species varies from six to eight (*Fig. 6*). The sequences that are exons in certain species may constitute introns in other species. The size of certain exons may vary due to the formation of new exon–intron borders. The size of the largest first exon of the *Xist *gene varies due to amplification and deletions of the tandem repeats within it and insertions/ deletions of taxon-specific mobile elements. Because of this variability, the length of *Xist *RN A in representatives of different orders may differ approximately twofold. The differences in the *Xist *gene in terms of RN A size, presence of exons, repeats, and mobile elements are believed to be attributable to its adaptation to functioning in the genome and to the X chromosome in each particular species.

The mouse XIC has two additional genes that encode nuclear RN A: *Tsix, *which is expressed from the antisense chain of the *Xist *gene, and *Xite *(X-inactivation intergenic transcriptional element). These genes control* Xist *expression during imprinted and random inactivation; they are involved in the counting of the number of X chromosomes per diploid autosomal set and choice of the future inactive X chromosome [66]. These genes are less conserved. Not all rodents possess the *Xite *gene; it has not been detected in humans [67]. Antisense transcription with respect to the *Xist *gene (similar to that for *Tsix*) has been detected in humans; however, it does not exhibit the same functions it does in mice [68, 69]. Thus, no conserved elements of XIC responsible for the functions of “counting” and “choice” have been found; hence, the functional elements of XIC, *Xist *regulation, and the inactivation process are at least partially species- specific [67].

In general, it can be noted that the genes of nuclear RN As involved in the inactivation process in XIC of eutherian mammals evolve very quickly. Their exon–intron structure and borders are changed; some noncoding RN As participating in the inactivation process are lost, while some others emerge during the evolution. Against this background, the replacement of the *Rsx* gene in marsupials by the *Xist *gene in eutherian mammals seems to be a trivial phenomenon, which properly complies with the general evolutionary trends of the inactivation process.

## COEVOLUTION OF the X CHROMOSOME AND THE X INACTIVATION PROCESS

**The X inactivation process limits the exchange of genetic material between the X chromosome and autosomes**

The evolution of mammalian sex chromosomes and X chromosome inactivation occur in an interrelated manner. The necessity of dosage compensation of X-linked genes emerged in mammals during the differentiation of sex chromosomes that had originally been a homologous autosomal pair. The process of X chromosome inactivation emerged after the Y chromosome started to lose homologues of the X chromosome genes and to accumulate the genes that participate in male gametogenesis as a result of recombination repression between the proto-X and proto-Y chromosomes [70]. The homology on the X and Y chromosomes was retained within a short region referred to as the pseudoautosomal region (PAR), which is required to ensure correct conjugation between the X and Y chromosomes during male meiosis. The PAR genes, which are homologous on the X and Y chromosomes, require no dosage compensation and avoid inactivation. The inactivation process presumably emerged in the common ancestor of marsupial and eutherian mammals on the X chromosome, which was compositionally close to the marsupial X chromosome. Further translocations of autosomal material to the ancestral X chromosome, which are observed in eutherian mammals, are supposed to have occurred in such a manner as not to disturb the dosage compensation. Otherwise, these rearrangements would have been eliminated by selection. It has been assumed that dosage compensation had not been disturbed when autosomal material had been added to the PAR of the X chromosome, followed by translocation to the PAR in the Y chromosome via recombination. At the initial stages, the autosomal genes newly added to the PAR in the X and Y chromosomes required no dosage compensation. Then, along with gene degradation in PAR on the Y chromosome, their homologues on the X chromosome became involved in the inactivation process. There were five sequential translocations on the mammalian X chromosome, resulting in the addition of autosomal genes to the ancestral X chromosome and the formation of younger evolutionary strata. In modern mammals, the lowest number of active homologues of the Y chromosomes has been retained in the most ancient (conserved) part of the X chromosome (*Fig. 1*), while the added regions contain more genes that avoid inactivation and have an active homologue on the Y chromosome [71]. Nevertheless, the eutherian X chromosome contains genes that avoid inactivation despite the fact that their homologue on the Y chromosome has been eliminated. Thus, the involvement of the genes in the inactivation process presumably takes some time; it appears that it does not take place immediately after the Y homologues are eliminated. Moreover, it has been noted that a twofold decrease in the amount of the product of one gene may have no adverse effects on a cell and the organism; hence, there is no need for gene dosage adjustment [72]. The dosage gene compensation in sex chromosomes seems to be aimed at maintaining the collective functions of the genes (e.g., the total protein concentration per cell), which depends on a number of expressible genes. Significant changes in the concentration of cytoplasmic proteins may disturb the ion concentration gradient on the cell membrane. Excess of protein products of X chromosome genes due to disturbance of inactivation results in the development of autoimmune diseases. Thus, disturbance of the collective gene functions may act as the driving force behind the evolution of dosage compensation.

An interesting solution to the problem of translocation of autosomal material to the X chromosome has been revealed in the common shrew *Sorex araneus *. Common shrews have not experienced the recombinational transfer of the translocated autosomal fragment to the Y chromosome; hence, the X chromosome in modern common shrews has two homologues: one corresponding to the ancestral Y chromosome (Y_1_), while the other one corresponds to the translocated autosome (Y_2_) [73]. The major part of the short arm of the X chromosome (original X) behaves as a typical eutherian X chromosome: it conjugates to the true Y chromosome during male meiosis and undergoes inactivation in female somatic cells. The added region, which occupies the long arm and the small pericentromeric region of the short arm, is identical to the autosome in terms of its behavior: it conjugates to Y_2_ and does not undergo inactivation.

**X chromosomes in eutherian mammals are enriched in LINE1 retrotransposons that participate in propagation and/or maintenance of the inactive status**

The autosomal genes linked to the inactivation center were found to be inactivated less efficiently as compared to X chromosome genes. An assumption has been put forward that the X chromosome has presumably accumulated specific sequences participating in propagation and/or maintenance of the inactive status. M.F. Lyon [74] has mentioned that this role can be played by LINE 1 retrotransposon, whose density on the mouse X chromosome is higher than that on autosomes. This hypothesis has been further supported by data obtained by an analysis of sequenced mammalian genomes. The LINE 1 content on the X chromosomes in mice, rats, and humans is twice as high as that on autosomes. LINE 1 are distributed rather uniformly along the eutherian X chromosome; their fraction is reduced only in the regions containing the genes that avoid inactivation [75, 76]. In the gray short-tailed opossum* M. domestica*, the fractions of LINE 1 localized in the X chromosome and autosomes do not significantly differ. This fact agrees with the data on incomplete and instable inactivation in marsupial mammals and demonstrates that an increased LINE 1 content is associated with their role in the inactivation process rather than being caused by the less efficient negative selection of LINE 1, due to the decrease in the frequency of meiotic recombinations of the X chromosome as compared to autosomes. The experimental data demonstrate that LINE 1 can participate in the arrangement of the chromosomal area of the inactive X chromosome; evolutionarily, the youngest LINE 1 are expressed on the inactivated X chromosome and promote propagation of the inactive status [77].

## CONCLUSIONS

Thus, it can be said that the process of X chromosome inactivation in marsupial and eutherian mammals has common epigenetic and, possibly, molecular mechanisms (*Fig. 2*). The key feature of the inactivation process in mammals, the coordinated gene repression at the level of the X chromosome, is presumably a result of the propagation of the nuclear noncoding RN A along it. However, the *Rsx *gene of nuclear noncoding RN A was replaced in eutherians during evolution by *Xist*, which is better, as compared to its ancestor, at attracting modifications, providing stable gene inactivation, to the X chromosome. The inactivation center with elements capable of counting and choosing the future active and inactive chromosomes was formed around the *Xist* gene, which made random repression of one of the two X chromosome possible. Furthermore, the formation of the more complete and stable inactivation in eutherians was promoted by the involvement of the mechanisms of DNA methylation in the maintenance of the inactive status and enrichment of the X chromosome in LINE 1 sequences, which increase the efficiency of propagation of the inactive state. Nevertheless, the evolution of X chromosome inactivation in mammals remains poorly studied. The hypotheses about its origin and evolution presented in this review are sometimes illogical and too speculative, since they are mainly based on phenomenological data, rather than on the knowledge of the mechanisms, which may differ even when being phenomenologically identical. Further research into the molecular and epigenetic mechanisms of this process could make it possible to better reconstruct the picture of the evolution of the dosage compensation system in mammals.

## Abbreviations

XIC: X inactivation center
PAR: pseudoautosomal region of the mammalian X chromosome
